# Calliterpenone, a natural plant growth promoter from a medicinal plant *Callicarpa macrophylla*, sustainably enhances the yield and productivity of crops

**DOI:** 10.3389/fpls.2022.960717

**Published:** 2022-09-26

**Authors:** Praveen Pandey, Shiv Shanker Pandey, Ashutosh Awasthi, Arpita Tripathi, Hemendra Pratap Singh, Anil Kumar Singh, Sudeep Tandon, Alok Kalra

**Affiliations:** ^1^Microbial Technology Department, CSIR-Central Institute of Medicinal and Aromatic Plants, Lucknow, India; ^2^Biotechnology Division, CSIR-Institute of Himalayan Bioresource Technology, Palampur, India; ^3^Faculty of Education, Teerthanker Mahaveer University, Moradabad, India; ^4^Biostatistics Department, CSIR-Central Institute of Medicinal and Aromatic Plants, Lucknow, India; ^5^Herbal and Medicinal Products Division, CSIR-Central Institute of Medicinal and Aromatic Plants, Lucknow, India; ^6^Process Chemistry and Chemical Engineering, CSIR-Central Institute of Medicinal and Aromatic Plants, Lucknow, India

**Keywords:** calliterpenone, plant growth regulators, IAA, ABA, yield contributing traits, sustainable crop production, medicinal plants

## Abstract

The global population is rising at an alarming rate, which is threatening food and nutritional security. Although chemical fertilizers and pesticides are important for achieving food security, their excessive usage critically affects soil health and adds up residues in the food chain. There is an increasing interest in identifying eco-friendly farm inputs that can improve crop productivity through sustainable agricultural practices. One of the most common approaches to reducing chemical inputs in agriculture is the use of plant growth regulators (PGRs). Here, we demonstrate the benefits of a natural and novel plant growth enhancer “calliterpenone,” isolated from *Callicarpa macrophylla*, a medicinal plant, for increasing crop productivity in six crops, *viz*., rice, wheat, potato, tomato, chickpea, and onion. Results revealed that the application of calliterpenone (foliar spraying or seed soaking) enhanced the yield of rice (28.89%), onion (20.63%), potato (37.17%), tomato (28.36%), and chickpea (26.08%) at 0.001 mM and of wheat (27.23%) at 0.01 mM concentrations in comparison to control. This enhancement in yield was reflected through improvements in its growth attributes, *viz*., spike length, tillers plant^−1^, seeds spike^−1^, plant height, and biomass. Furthermore, the exogenous application of calliterpenone could increase the endogenous level of indole-3-acetic acid (IAA) in all tested crops and decrease the content of abscisic acid (ABA) in a few. Trials conducted at farmers' fields showed an overall ~12% increase in rice yield (mean of 11 farmers' fields ranging from 3.48 to 19.63%) and ~10% increase in wheat yield (ranging from 3.91 to 17.51%). The 0.001 mM of calliterpenone was the best effective dose for most crops except wheat, where a concentration of 0.01 mM was found to be the most optimal. This study indicates that calliterpenone is a natural plant growth promoter that can be used in boosting the yields of multiple crops and would be an important input component of organic farming.

## Introduction

The green revolution considerably enhanced food grain production by employing high-yielding varieties (HYVs), adequate fertilizers, and modern farming practices (Swaminathan and Bhavani, 2013). However, since agricultural land resources are shrinking day by day and the population is rapidly increasing, it is projected to grow by over 8 billion by 2025 (Hinrichsen and Rowley, 1999). Hence, an urgent need is felt to enhance crop productivity to sustain this ever-increasing population. Although chemical fertilizers and pesticides are important for achieving food security, their excessive usage critically affects soil health and environmental quality (Tilman, 1998). Therefore, an eco-friendly and sustainable approach must be explored to meet the increasing population demand from a limited cultivation area. One of the common approaches to reducing chemical inputs in agriculture is the use of plant growth regulators (PGRs) such as gibberellins, auxins, and cytokinins (Masroor et al., 2006; Tiwari et al., 2011; Giannakoula et al., 2012; Choudhury et al., 2013; Rastogi et al., 2013; Kurubar et al., 2017; Zhang et al., 2017; McMillan et al., 2020; Chen et al., 2021). PGRs are important for enhancing plant growth and productivity in intensive agriculture, especially for high-value crops. Gibberellins (especially gibberellic acid) are important PGRs used for increasing the economic yields of various crops. These have been found effective in rice (Gavino et al., 2008; Tiwari et al., 2011; Prajapati et al., 2021), wheat (Pavlista et al., 2014; Amram et al., 2015; Zhang et al., 2017; Dawar et al., 2021), potato (Thornton et al., 2013), tomato (Choudhury et al., 2013), cotton (Copur et al., 2010), sweet pepper (Maboko and DuPlooy, 2015), *Pyrethrum* (Singh et al., 2011), peppermint (Khanam and Mohammad, 2017), onion (Ghani et al., 2021), and chickpea (Rafique et al., 2021). However, the higher cost of gibberellins has restricted their use to high-value crops only. CSIR-Central Institute of Medicinal and Aromatic Plants (CSIR-CIMAP) isolated “calliterpenone”, a novel and natural plant growth-promoter from *Callicarpa macrophylla* (Singh et al., 2004, 2009), which could be useful in increasing the growth and yield of several crop plants.

*Callicarpa macrophylla* Vahl (Family Verbenaceae) is a valuable medicinal plant effective for treating many diseases such as fever, diarrhea, diabetes, tumor, polydipsia, and dysentery (Pandey et al., 2014). This erect shrub is widely distributed over sub-Himalayan and Indo-Gangetic regions of India (Chopra et al., 1986; Manandhar, 2002). About 20 species of *Callicarpa* are found in South Asia and China (Jones and Kinghorn, 2008). Various compounds including carbohydrates, amino acids, lipids, flavonoids, diterpenes, benzenoids, phytosterols, triterpenes, phenylpropanoids, and sesquiterpenes have been reported from *Callicarpa* (Tellez et al., 2000).

**Figure 1:**
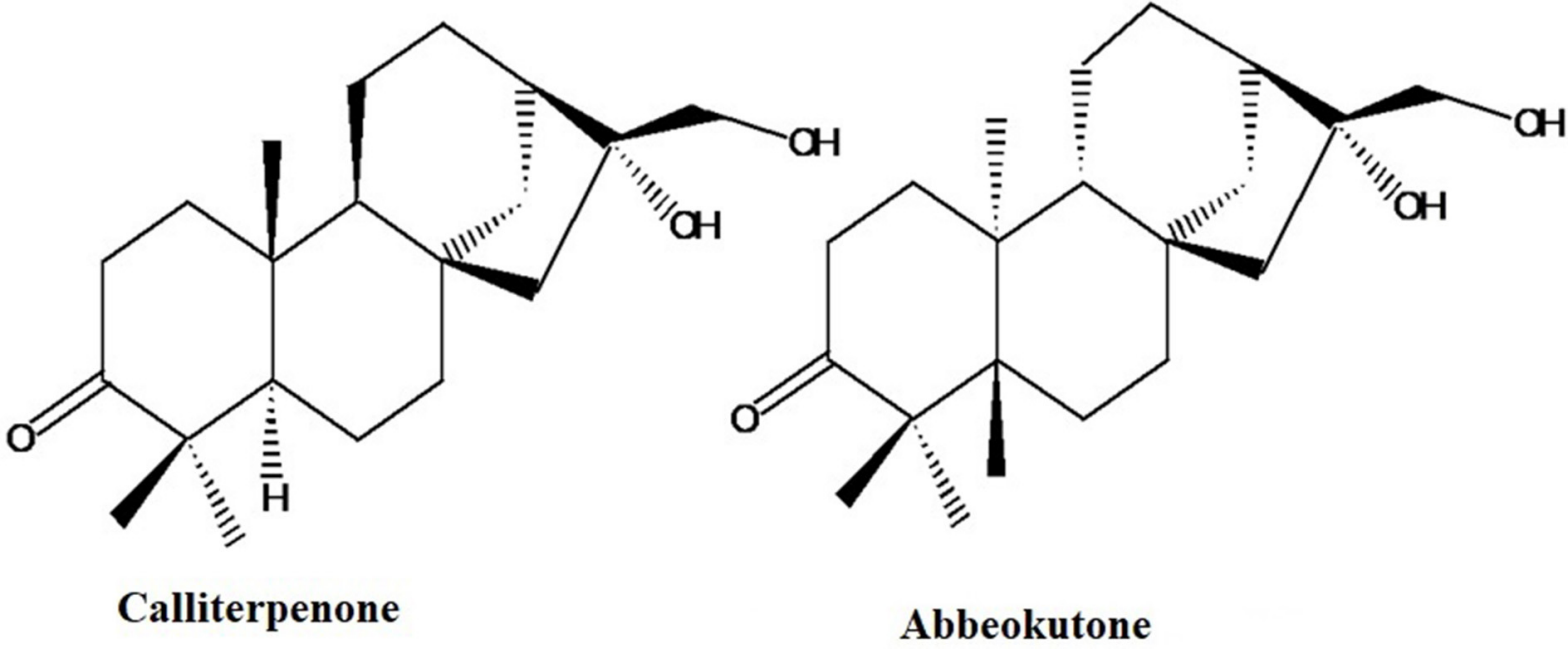
Structure of calliterpenone and abbeokutone.

Calliterpenone (16α, 17-dihidroxy phyllocladane-3-one) (Figure 1), has a similar substitution pattern similar to the ent-kaurenoid compound “abbeokutone” (16α, 17-dihidroxy kaurane-3-one), the precursor of gibberellins in the biosynthetic pathway (Liu et al., 2003; Bottini et al., 2004). The structural relationship of calliterpenone to gibberellic acid prompted us to evaluate its plant growth-promoting activities. It was found that calliterpenone and its mono-acetate induced better plant growth than well-established PGRs such as gibberellic acid and many cytokinins (Singh et al., 2004, 2011; Goel et al., 2007; Bose et al., 2013). Also, as calliterpenone is obtained from a natural resource, this could be a beneficial product for organic farming. The organic food market's projected compound annual growth rate (CAGR) is likely to grow by 16.15% during 2017–2021 globally due to rising incomes and growing consumer awareness concerning the health benefits of consuming organic food. The projections for the growth of organic farm produce in India are about 25%. The market for PGRs was around USD 2.11 billion in 2017 and may grow to a value of USD 2.93 billion by 2022, at a CAGR of 6.8% (TechSci Research, 2017).

It has also been found that the calliterpenone has the potential to alleviate the growth-retarding effects of allelochemicals (e.g., *Artemisia annua* seed extract) (Anaya et al., 2003; Singh et al., 2004) and promote plant growth. Calliterpenone improved the yield of flowers and the content of pyrethrin in *Pyrethrum* flowers (Singh et al., 2011). In *Mentha arvensis*, calliterpenone has been found to induce sprouting, increase branching, herb yield (Haider et al., 2009), oil content, and trichome density (Bose et al., 2013). Calliterpenone could also enhance the number of cells of *Bacillus, Rhizobium*, and *Cyanobacteria* (Kalra et al., 2010; Maji et al., 2013; Patel et al., 2014) in the growth medium and significantly improved the growth as well as multiplication of *in vitro* shoot cultures of *Rauwolfia serpentina* (Goel et al., 2007). Recently, Zafar and Sangwan (2019) studied physiological and chemical changes related to the growth-enhancing effects of calliterpenone on *Mentha arvensis*. They found that application of calliterpenone significantly increases chlorophyll content, total sugars, protein content, moisture content, and activity of invertase enzyme, along with considerable enhancement in phenotypic growth parameters (number of branches, plant height, internodal length, and the total numbers of fresh sprouting). These findings indicate that calliterpenone is an efficient and economic plant growth promoter. Considering the importance of calliterpenone, efficient and economically viable process technology for isolation of calliterpenone from leaves of *C. macrophylla* was developed and patented worldwide (Singh et al., 2004, 2009, 2016). Although its plant growth-promoting effect on medicinal and aromatic plants is well known, no information is available on its efficacy in food crops. This study is our effort to establish its growth-promoting potential in major groups of field crops, *viz*., cereals, vegetables, and pulses, which will surely help in its commercialization as plant growth promoters. Therefore, this study was undertaken to evaluate the effects of calliterpenone on some agriculturally important food crops to enhance their yield and productivity in an eco-friendly manner without increasing the additional chemical load on soil.

## Materials and methods

### Site description

The site of this study was the experimental farm of CSIR-Central Institute of Medicinal and Aromatic Plants, Lucknow, India, geographically located at 26.05°N latitude, 80.05°E longitude, and an altitude of 120 m above mean sea level, experiencing a semiarid subtropical climate with an annual rainfall of 900 mm, with hot summers and cold winters being the main characteristics of the region. The soil characteristics of the field are sandy loam in texture with pH 7.95, EC 0.18 dS m^−1^, organic carbon 0.40%, nitrogen 162.75 kg ha^−1^, phosphorus 15.02 kg ha^−1^, and potassium 161.12 kg ha^−1^.

The farmers' field trials were conducted in the fields of 11 farmers in the districts of Lucknow (26.05°N, 80.05°E), Sitapur (27.58°N, 80.66°E), and Raebareli (26.23°N, 81.24°E) in Uttar Pradesh, India.

### Plant materials and experimental design

Field experiments were conducted on food crops, *viz*., wheat (*Triticum aestivum*), rice (*Oryza sativa)* potato (*Solanum tuberosum)*, chickpea (*Cicer arietinum*), tomato (*Solanum lycopersicum*), and onion (*Allium cepa*). The experimental material consisted of ten treatment combinations of each crop (Table 1) arranged in an RBD (randomized block design) with three replications in two consecutive crop seasons during 2016–2018. The individual plot size of each treatment and spacing between rows and plants varied from crop to crop. For tomato, potato, and onion, the plot size was 4 m^2^ (4 rows of 2 m, with 50 cm spacing between rows). However, plant-to-plant spacings were 50 cm, 15 cm, and 10 cm for tomato, potato, and onion, respectively. In the case of chickpea, 4 m^2^ plot size consisted of 5 rows of 2 m, spaced 40 cm apart between rows and 10 cm apart between the plants, while in rice, 7 m^2^ plots (10 rows of 3.5 m with a spacing of 20 and 10 cm between rows and plants, respectively) and in wheat, 3.5 m^2^ plots (3.5 m row length of 5 rows having 20 cm spacing between rows and 5 cm between plants). Crop varieties used for the experiments were Omkar-283 (tomato), Chipsona (potato), Punjab selection (onion), DCP-92-3 (chickpea), Tilakchandan (rice), and HD-2967 (wheat). For trials conducted at farmers' fields, the plot size of each farmer was 500 m^2^. The doses of fertilizers (N:P:K kg ha^−1^) applied in different crops were rice (120:60:40), wheat (150:60:40), tomato (180:100:60), potato (120:80:80), onion (100:50:50), and chickpea (20:40:20). The total amount of potassium and phosphorus were applied as basal dose at the time of planting through muriate of potash (MOP) and single super phosphate (SSP), respectively. Nitrogen was supplemented through urea in three splits, i.e., half as basal dose at the time of planting, and the rest of nitrogen was top-dressed in two equal split doses in 30- and 55-days old crops. The other agronomic practices, *viz*., irrigation, weeding, insecticides, and pesticides, were adopted as and when required to have a good crop stand.

**Table 1:** Detail of treatments used in various crops.

**Treatments**	**Calliterpenone concentration and application method**	**Crops**					
		**Rice**	**Wheat**	**Tomato**	**Potato**	**Onion**	**Chickpea**
Control	Treated with water	✓	✓	✓	✓	✓	✓
ST1	Seed treatment with 0.1 mM	✓	✓	✓	✓		✓
ST2	Seed treatment with 0.01 mM	✓	✓	✓	✓		✓
ST3	Seed treatment with 0.001 mM	✓	✓	✓	✓		✓
STA1	Seed treatment + 1 spray with 0.1 mM		✓		✓		✓
STA2	Seed treatment + 1 spray with 0.01 mM		✓		✓		✓
STA3	Seed treatment + 1 spray with 0.001 mM		✓		✓		✓
STB1	Seed treatment + 2 spray with 0.1 mM		✓		✓		
STB2	Seed treatment + 2 spray with 0.01 mM		✓		✓		
STB3	Seed treatment + 2 spray with 0.001 mM		✓		✓		
SRT1	Seed + root treatment with 0.1 mM	✓					
SRT2	Seed + root treatment with 0.01 mM	✓					
SRT3	Seed + root treatment with 0.001 mM	✓					
SRTA1	Seed + root treatment + 1 spray with 0.1 mM	✓		✓			
SRTA2	Seed + root treatment + 1 spray with 0.01 mM	✓		✓			
SRTA3	Seed + root treatment + 1 spray with 0.001 mM	✓		✓			
SRTB1	Seed + root treatment + 2 spray with 0.1 mM			✓			
SRTB2	Seed + root treatment + 2 spray with 0.01 mM			✓			
SRTB3	Seed + root treatment + 2 spray with 0.001 mM			✓			
SP1	Spray with 0.1 mM						✓
SP2	Spray with 0.01 mM						✓
SP3	Spray with 0.001 mM						✓
RT1	Root treatment with 0.1 mM					✓	
RT2	Root treatment with 0.01 mM					✓	
RT3	Root treatment with 0.001 mM					✓	
RTA1	Root treatment + 1 spray with 0.1 mM					✓	
RTA2	Root treatment + 1 spray with 0.01 mM					✓	
RTA3	Root treatment + 1 spray with 0.001 mM					✓	
RTB1	Root treatment + 2 spray with 0.1 mM					✓	
RTB2	Root treatment + 2 spray with 0.01 mM					✓	
RTB3	Root treatment + 2 spray with 0.001 mM					✓	

### Extraction and isolation of calliterpenone

Calliterpenone was extracted from the leaves of the *C. macrophylla* plant as described earlier (Singh et al., 2009, 2016). Two hundred grams of cleaned and dried leaves were extracted with 6 L of 1% alkaline water for 3 h in a Clevenger-like glass apparatus (10 L capacity). The resulting extract was reduced to about one-third volume and was further extracted in dichloromethane (3 × 200 ml). The pooled dichloromethane extract was concentrated to a viscous residue. The residue was purified by celite filtration, i.e., absorbed over 2 g celite and passed through a bed prepared from 20 g celite in a vacuum filtration funnel fitted with a glass sintered disc. It was first eluted with 10% ethyl acetate in hexane (600 ml) and then with 50% ethyl acetate in hexane (1 L). This 1 L of 50% ethyl acetate hexane elution was dried under vacuum to produce calliterpenone (0.46%), having 84% purity as estimated by HPLC as described by Verma et al. (2009). For pilot scale extraction, 15 kg of dried leaves were processed similarly in a stainless still distillation tank, and for extraction, dichloromethane continuous solvent–solvent extraction technology was used. It was further purified by column chromatography.

### Dose and method of calliterpenone treatment

A stock solution (0.1 mM) of calliterpenone was made by dissolving 3.2 mg of calliterpenone in 1 ml of absolute alcohol followed by 100 ml of water. To prepare 0.01 mM and 0.001 mM solutions of calliterpenone, 10 ml of stock solution was taken in volumetric flasks, and the volume was made up to 100 ml and 1,000 ml by adding distilled water, respectively. Three different concentrations (0.1, 0.01, and 0.001 mM) of calliterpenone were used; seed soaking (6 h), root treatment of seedlings before transplanting (3 h), and foliar spraying at 30 days and 60 days after planting (DAP). However, the methods of application varied from crop to crop (Table 1). The foliar application was carried out at 30 DAP and 60 DAP by spraying calliterpenone (~0.25 L/m^2^) on plants uniformly to the point of run-off using a Knapsack sprayer with constant flow. Utmost care was taken while spraying to ensure that no run-off from the leaves reaches the soil that may possibly affect soil microbial activity. Control plots were treated with the same volume of water. The selection of various doses of calliterpenone is based on our initial evaluation of doses of calliterpenone for their growth-promoting activities, wherein the most suitable concentrations were recorded as 0.001–0.1 mM (w/v basis) while increasing the concentration growth inhibitory activities were observed in some instances (Bagchi et al., 1997; Singh et al., 2004). Based on the results of the main field experiments of this study, 0.001 mM dose for rice and 0.01 mM for wheat were selected for assessment of its effects on farmers' fields trials; the applications were made at the time of sowing as seed treatment followed by a foliar spray at 30 DAP.

### Data collection on yield and yield attributing traits

Data were collected for each treatment from 10 randomly selected representative plants avoiding border row plants for plant height (cm), tillers plant^−1^, panicle length or spike length (cm), seeds panicle^−1^ or seeds spike^−1^, grain yield plant^−1^ (g), grain yield plot^−1^ (kg), biological yield plant^−1^ (g), harvest index (%), and test weight (g). However, data on days to 50% flowering were recorded on a plot basis; during the crop standing stage, plants were monitored daily to observe the initiation of the flowering, and when 50% of plants in each plot flowered the date was noted and calculated the number of days from the date of sowing. At the time of physiological maturity, plant height and number of tillers plant^−1^ were measured; the number of tillers of 10 plants were counted and averaged to calculate tillers plant^−1^. After harvesting, plants were separated into straw and panicles and panicle length was measured. The panicles were hand-threshed and the total numbers of seeds were counted and averaged to determine the number of seeds panicle^−1^. The ratio of grain yield to total dry matter of the plant (biological yield) was considered as harvest index and expressed in percentage. It was calculated by the following formula given by Donald and Hamblin (1976):


$$\begin{array}{l} \text{Harvest index }\left(\%\right)=\frac{\text{Grain yield}}{\text{Biological yield}}\text{ x }100 \end{array}$$

To calculate test weight filled seeds were separated from unfilled seeds and 1,000 seeds were counted and weighed. In order to calculate biological yield plant^−1^, the whole plants above the ground level were harvested, sun-dried, and weighed; on the other hand, to determine grain yield plant^−1^, the total quantity of the grains obtained after threshing was cleaned and weighed. For calculating the yield of farmers' fields, 10 samples from treatment and control plots were harvested using 1 × 1 m quadrat excluding border rows and averaged.

### Standard solution and sample preparation for residue analysis

For residue analysis, plant samples (rice leaves, straw, grains, husk, and soil) were collected from the plants on which morphological data were recorded. Samples (in triplicate) that received maximum concentration treatment were considered for the study. After harvesting the plants, straws, leaves, and grains were separated. Furthermore, husks were removed from grains by hand hulling. Samples were grounded to a fine powder, and a total of 1.0 g of the powdered tissue was extracted in 10 ml of 2% alkaline water (200 mg NaOH in 10 ml water) for 2 h, followed by portioning of the extracts using dichloromethane. The dichloromethane extracts were then dried and later dissolved in methanol. Before HPLC injection, solutions were filtered through 0.45 μm membranes (Millipore, Billerica, MA). For extraction of calliterpenone, the HPLC analysis was performed as per the method described by our group (Verma et al., 2009) using an LC-10A HPLC system (Shimadzu, Japan) with acetonitrile:water (45:55) as mobile phase and detection was made at 220 nm using a Waters Spherisorb ODS-2 column (250 × 4.6 mm I.D., 10 μm).

### Measurement of endogenous indole-3-acetic acid (IAA) and abscisic acid (ABA) content

The quantitative determinations of ABA and IAA contents were carried out using Phytodetek-IAA and ABA immunoassay kit (Agdia, Elkhart, IN) following the manufacturer's protocol. Phytohormone extraction from leaf samples of different crops was performed as described by Barnawal et al. (2016). Leaf samples frozen in liquid nitrogen were grounded to a fine powder. A total of 0.5 g of the powdered leaf tissue in 8 ml of extraction solution consisting of methanol (80%), butylated hydroxytoluene (BHT,100 mg L^−1^), and citric acid monohydrate (0.5 g L^−1^) was continuously stirred in the dark at 4°C for overnight. The solution was then centrifuged at 4°C for 20 min at 1,000 g. Later, the supernatant was dried under a vacuum. The dry residue was then dissolved in a solution containing 900 μl of Tris-buffered saline [T.B.S. (pH 7.8)] and 100 μL of methanol (100%). The concentrations of ABA and IAA in the filtrate were determined with ABA/IAA enzyme immunoassay test kit and expressed based on fresh weight (FW).

### Statistical analysis

The data were compiled using mean values of randomly selected plants in each treatment and employed for statistical analyses. The analysis of variance (ANOVA) for RBD was performed as per the methodology suggested by Panse and Sukhatme (1985). Treatments were statistically compared through critical differences at the *P* ≤ 0.05 level of significance by Fisher's *F*-test.

## Results

The experimental findings concerning various growth and yield parameters in tested crops, including cereals (rice, wheat), vegetables (tomato, potato, onion), and pulse crop (chickpea), have been illustrated in the following sections:

### Effect of calliterpenone on plant growth, yield, and yield contributing traits in rice

**Figure 2:**
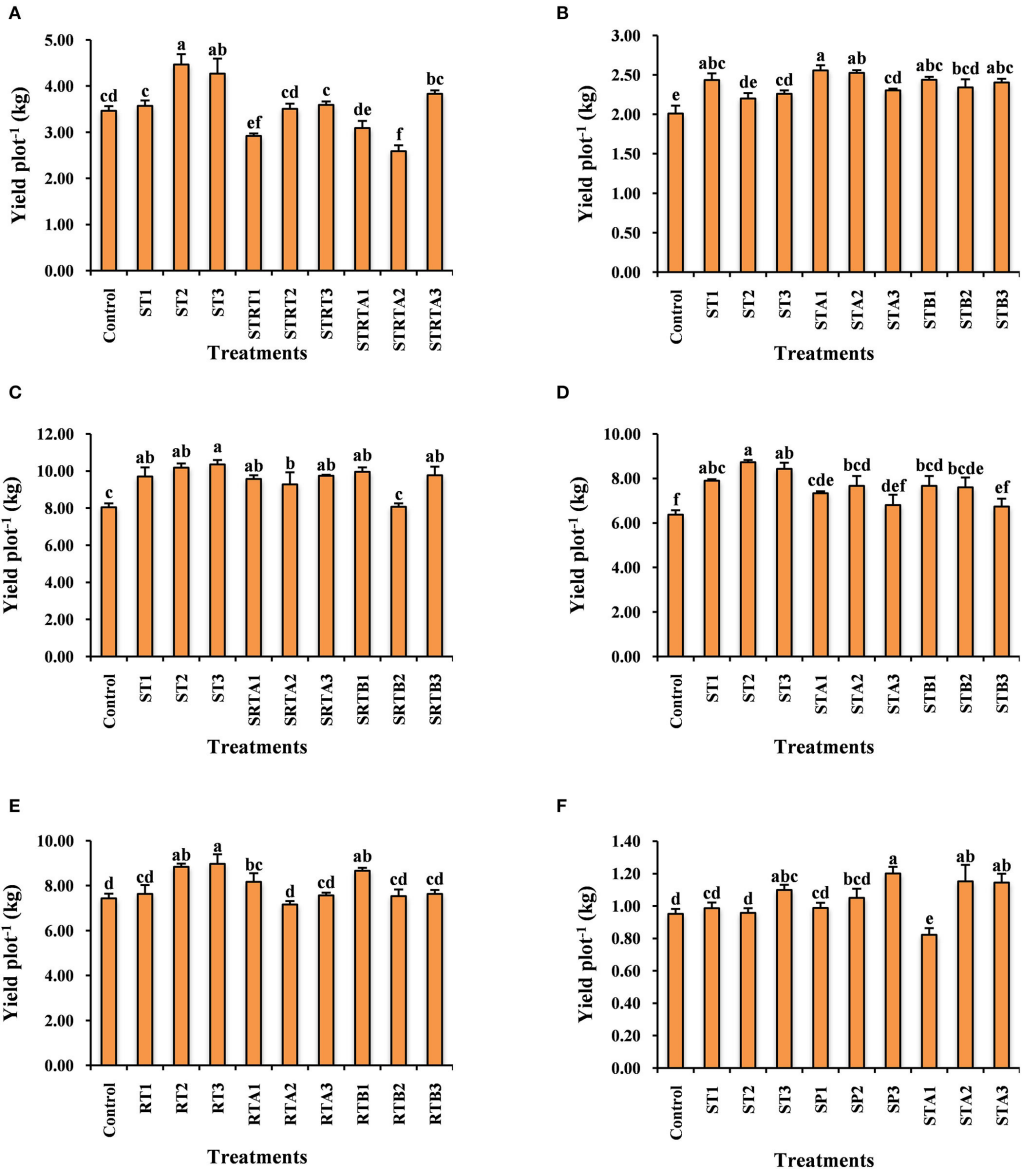
Effect of calliterpenone on yield enhancement in rice **(A)**, wheat **(B)**, tomato **(C)**, potato **(D)**, onion **(E)**, and chickpea **(F)**. Statistical analysis was carried out taking means of three replicates; different letters above bars showed significant differences as compared to control through critical difference (*P* ≤ 0.05). ST1, seed treatment with 0.1 mM; ST2, seed treatment with 0.01 mM; ST3, seed treatment with 0.001 mM; STA1, seed treatment + 1 spray with 0.1 mM; STA2, seed treatment + 1 spray with 0.01 mM; STA3, seed treatment + 1 spray with 0.001 mM; STB1, seed treatment + 2 spray with 0.1 mM; STB2, seed treatment + 2 spray with 0.01 mM; STB3, seed treatment + 2 spray with 0.001 mM; SRT1, seed treatment + root treatment with 0.1 mM; SRT2, seed treatment + root treatment with 0.01 mM; SRT3, seed treatment + root treatment with 0.001 mM; SRTA1, seed treatment + root treatment + 1 spray with 0.1 mM; SRTA2, seed treatment + root treatment + 1 spray with 0.01 mM; SRTA3, seed treatment + root treatment + 1 spray with 0.001 mM; SRTB1, seed treatment + root treatment + 2 spray with 0.1 mM; SRTB2, seed treatment + root treatment + 2 spray with 0.01 mM; SRTB3, seed treatment + root treatment + 2 spray with 0.001 mM; SP1, spray with 0.1 mM; SP2, spray with 0.01 mM; SP3, spray with 0.001 mM; RT1, root treatment with 0.1 mM; RT2, root treatment with 0.01 mM; RT3, root treatment with 0.001 mM; RTA1, root treatment + 1 spray with 0.1 mM; RTA2, root treatment + 1 spray with 0.01 mM; RTA3, root treatment + 1 spray with 0.001 mM; RTB1, root treatment + 2 spray with 0.1 mM; RTB2, root treatment + 2 spray with 0.01 mM; RTB3, root treatment + 2 spray with 0.001 mM.

The data relating to the effect of calliterpenone on yield contributing traits and yield in rice is given in Table 2 and Figure 2A, and the percentage of the yield increase over control in Supplementary Table S1. All the characteristics were significantly different except for days to 50% flowering and test weight. A maximum yield increase of 28.89% was observed in treatment ST2 (seed treatment with 0.01 mM), followed by 23.26% in ST3 (seed treatment with 0.001 mM), significantly higher than control. However, treatments like SRTA2 (seed treatment + root treatment + 1 spray with 0.01 mM), SRT1 (seed treatment + root treatment with 0.1 mM), and SRTA1 (seed treatment + root treatment + 1 spray with 0.1 mM) showed significantly lower yields than control, i.e., – 25.28, – 15.71, and – 10.78%, respectively.

**Table 2:** Effect of calliterpenone on yield and yield contributing traits in rice.

**Treatments**		**Days to 50% flowering**	**Plant height (cm)**	**Tillers plant^−1^**	**Panicle length (cm)**	**Seeds panicle^−1^**	**Biological yield plant^−1^ (g)**	**Grain yield plant^−1^(g)**	**Harvest index (%)**	**Test weight (g)**
Control		118.33	175.33	9.93	28.87	162.17	80.60	21.36	26.57	18.67
ST1		119.33	177.17	9.50	29.22	158.70	83.40	23.11	27.70	18.97
ST2		118.67	177.97	10.57	29.50	172.80	84.40	24.56	29.13	19.07
ST3		118.67	173.23	10.00	29.77	175.53	73.87	23.59	31.95	18.70
SRT1		118.00	178.13	10.50	29.77	168.67	99.27	23.16	23.54	19.00
SRT2		118.33	176.47	10.60	29.52	168.87	84.97	23.44	27.53	19.10
SRT3		117.67	182.33	10.43	29.77	162.00	97.33	27.30	28.25	18.33
SRTA1		118.33	175.17	9.47	28.70	158.10	101.97	27.51	26.96	18.53
SRTA2		118.67	176.90	8.77	29.43	163.90	89.57	24.83	27.72	19.30
SRTA3		119.33	178.60	9.77	30.51	169.87	82.07	20.33	25.13	18.17
F ratio		1.19	3.41	4.00	4.61	4.73	3.48	3.85	2.76	0.86
F prob.		0.36	0.01	0.01	0.00	0.02	0.01	0.01	0.03	0.58
C.V.		0.70	1.29	5.20	1.40	2.85	9.62	8.40	8.54	3.63
C.D. at 5%		1.72	3.93	0.89	0.71	8.12	14.49	3.45	4.02	1.18
S.E.M.		0.52	1.73	0.42	0.35	4.14	6.58	1.65	1.68	0.37
Range	Min	117.67	173.23	8.77	28.70	158.10	73.87	20.33	23.54	18.17
	Max	119.33	182.33	10.60	30.51	175.53	101.97	27.51	31.95	19.30

C.V., coefficient of variation; C.D., critical difference; S.E.M., standard error of mean. Other abbreviations are as shown in Table 1.

Early flowering is a desirable characteristic. The days to 50% flowering ranged from 117.67 to 119.33 days, and there were no significant differences among treatments. Treatment SRT3 (182.33 cm) followed by SRTA3 (seed treatment + root treatment + 1 spray with 0.001 mM) with 178.60 cm plants resulted in significantly taller plants than control. However, ST3-treated plants were a little shorter (173.23 cm) than the control (175.33 cm).

More effective tillers, large panicles, and the number of seeds panicle^−1^ are generally responsible for higher yields in rice. SRT2 (10.60) followed by ST2 (10.57), SRT1 (10.50), and SRT3 (10.43) led to higher tillering, while SRTA2 (8.77), SRTA1 (9.47), SRTA3 (9.77), and ST1 (9.50) had fewer tillers than control (9.93). The panicle size was significantly larger in plants receiving the treatments such as SRTA3 (30.51 cm), ST3 (29.77 cm), SRT1 (29.77 cm), SRT3 (29.77 cm), SRT2 (29.52 cm), and ST2 (29.50 cm) than the control (28.87 cm). The maximum number of seeds panicle^−1^ was observed in ST3 (175.33) followed by ST2 (172.80), SRTA3 (169.87), and SRT2 (168.87), which were significantly higher than in control (162.17). However, treatments SRTA1 with 158.10 and ST1 (seed treatment with 0.1 mM) with 158.70 produced fewer seeds panicle^−1^ than in control.

Maximum and significantly higher biological yield plant^−1^ was observed in SRTA1 (101.97 g), followed by SRT1 (99.27 g), and SRT3 (97.33 g). However, ST3 (73.87 g) had a lower biological yield plant^−1^ than control (80.60 g). Grain yield plant^−1^ was maximum in SRTA1 (27.51 g), followed by SRT3 (27.30 g), SRTA2 (24.83 g), and ST2 (24.56 g), significantly higher than control (21.36 g). However, SRTA3 (20.33 g) resulted in minimum grain yield plant^−1^.

Maximum harvest index was recorded in ST3 (31.95%) followed by ST2 (29.13%), SRT3 (28.25%), SRTA2 (27.72%), ST1 (27.70%), and SRT2 (27.53%), significantly higher than control (26.57%). However, SRT1 (23.54%) had a minimum harvest index. In the case of test weight, there were no significant differences among treatments.

### Effect of calliterpenone on plant growth, yield, and yield contributing traits in wheat

The data relating to the effect of calliterpenone on yield and yield contributing traits in wheat is presented in Table 3 and Figure 2B, and the percent yield increase over control in Supplementary Table S1. ANOVA revealed significant differences for most of the characters, except harvest index and test weight. Maximum yield increase over control 27.23% was observed in STA1 (seed treatment + 1 spray with 0.1 mM) followed by 25.71% in STA2 (seed treatment + 1 spray with 0.01 mM), 21.29% in STB1 (seed treatment + 2 spray with 0.1 mM), 21.16% in ST1 (seed treatment with 0.1 mM), 19.57% in STB3 (seed treatment + 2 spray with 0.001 mM), 16.59% in STB2 (seed treatment + 2 spray with 0.01 mM), 14.58% in STA3 (seed treatment + 1 spray with 0.001 mM), and 12.50% in ST3 (seed treatment with 0.001 mM).

**Table 3:** Effect of calliterpenone on yield and yield contributing traits in wheat.

**Treatments**		**Days to 50% flowering**	**Plant height (cm)**	**Tillers plant^−1^**	**Spike length (cm)**	**Seeds spike^−1^**	**Biological yield plant^−1^(g)**	**Grain yield plant^−1^(g)**	**Harvest index (%)**	**Test weight (g)**
Control		70.67	104.47	4.60	9.65	52.60	27.52	8.73	31.74	44.78
ST1		69.33	104.33	4.90	10.27	57.53	32.97	10.88	32.93	43.08
ST2		70.33	108.53	4.97	10.85	53.93	29.74	9.67	32.46	43.60
ST3		70.67	106.83	4.83	10.78	55.60	30.02	10.00	33.29	45.04
STA1		71.00	108.67	4.43	10.00	57.73	32.11	10.54	32.71	42.89
STA2		71.00	104.90	4.83	10.82	58.67	32.27	10.95	34.03	43.85
STA3		71.00	102.47	4.80	10.87	46.33	30.18	9.91	32.83	44.38
STB1		70.33	104.87	4.83	10.47	48.20	28.29	8.49	30.08	42.31
STB2		70.67	104.00	4.57	10.27	52.20	29.83	8.75	29.32	43.64
STB3		71.00	107.43	4.60	10.30	51.47	28.94	8.93	30.94	42.61
F ratio		3.60	10.52	4.95	5.76	4.03	2.61	2.52	1.01	0.90
F prob.		0.01	0.00	0.00	0.00	0.01	0.04	0.05	0.47	0.54
C.V.		0.67	1.05	2.85	2.83	6.62	6.31	10.39	8.05	3.84
C.D. at 5%		0.81	1.91	0.23	0.51	6.07	3.27	1.73	4.75	2.87
S.E.M.		0.36	1.28	0.12	0.27	2.82	1.42	0.73	1.46	0.11
Range	Min	69.33	102.47	4.43	9.65	46.33	27.52	8.49	29.32	42.31
	Max	71.00	108.67	4.97	10.87	58.67	32.97	10.95	34.03	45.04

C.V., coefficient of variation; C.D., critical difference; S.E.M., standard error of mean; Other abbreviations are as shown in Table 1.

The treatment ST1 (seed treatment with 0.1 mM) induced early flowering (69.33 days) than control (70.67 days). On the other hand, STA1 (seed treatment + 1 spray with 0.1 mM), STA2 (seed treatment + 1 spray with 0.01 mM), STA3 (seed treatment + 1 spray with 0.001 mM), and STB3 (seed treatment + 2 spray with 0.001 mM) resulted in late flowering (71.00 days) than control. Maximum plant height of 108.67 cm was recorded in STA1, followed by 108.53 cm in ST2 (seed treatment with 0.01 mM), 107.43 cm in STB2, and 106.83 cm in ST3, which were significantly higher than control (104.47 cm). The plant height was recorded the least in STA3 (102.89 cm).

Significantly higher tillering over control was recorded in ST2 (4.97), ST1 (4.90), STB1 (4.83), ST3 (4.83), STA2 (4.83), and STA3 (4.80). All the treatments resulted in significantly larger spikes than control (9.65 cm) except STA1 (10.00 cm). The length of the spike was observed to be maximum in STA3 (10.87 cm) followed by ST2 (10.85 cm), STA2 (10.82 cm), ST3 (10.78 cm), STB1 (10.47 cm), STB3 (10.30 cm), ST1 (10.27 cm), and STB2 (10.27 cm). The treatments STA2 (58.67) followed by STA1 (57.73) led to a significantly more number of seeds spike^−1^ than the control (52.60). However, a significantly lower number of seeds spike^−1^ were recorded in STA3 (46.33).

The treatments ST1 (32.97 g), STA2 (32.27 g), and STA1 (32.11 g) resulted in significantly higher biological yield plant^−1^ than control (27.52 g). The maximum grain yield plant^−1^ was recorded in STA2 (10.95 g), followed by ST1 (10.88 g) and STA1 (10.54 g). However, lower grain yield plant^−1^ than control (8.73 g) was recorded in STB1 (8.49 g). In the context of harvest index and test weight, it was observed that there were no significant differences among treatments.

### Effect of calliterpenone on the yield of vegetable crops tomato, potato, and onion

All the treatments in tomatoes (Figure 2C, Supplementary Table S1) resulted in significantly greater yields than control, except SRTB2 (seed treatment + root treatment + 2 sprays with 0.01 mM). The maximum yield increase of 28.63% was observed in ST3 followed by 26.59% in ST2, 23.77% in SRTB1 (seed treatment + root treatment + 2 spray with 0.1 mM), 21.39% in SRTB3 (seed treatment + root treatment + 2 spray with 0.001 mM), 21.10% in SRTA3, 20.55% in ST1, 18.94% in SRTA1 and 15.34% in SRTA2.

Most of the treatments showed significantly higher yields than the control, except STA3 and STB3. The maximum yield increase in potato (Figure 2D, Supplementary Table S1) was noticed in ST2 (37.17%) followed by ST3 (32.46%), ST1 (24.46%), STA2 (20.42%), STA1 (15.18%), STB1 (13.86%), and STB2 (12.87%).

In the case of onion (Figure 2E, Supplementary Table S1), considerably higher yields over control were observed in RT3 (root treatment with 0.001 mM) (20.63%) followed by RT2 (root treatment with 0.01 mM) (18.83%) and RTB1 (root treatment + 2 spray with 0.1 mM) (16.59%).

### Effect of calliterpenone on a pulse crop, chickpea

Chickpea is the most important pulse crop; however, its productivity is relatively low compared to cereals. With the use of calliterpenone, significantly higher yields (Figure 2F, Supplementary Table S1) were observed in treatments SP3 (spray with 0.001 mM) (26.08%) followed by STA2 (21.02%), STA3 (20.21%), ST3 (15.35%), and SP2 (spray with 0.01 mM) (10.35%).

### Effect of calliterpenone on endogenous IAA and ABA contents

**Figure 3:**
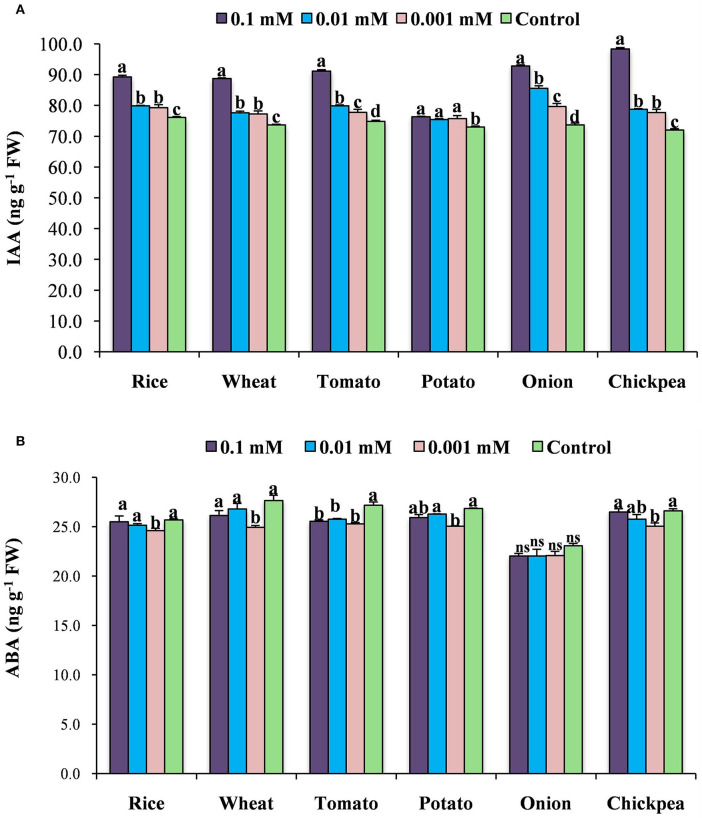
Effect of calliterpenone on endogenous IAA content **(A)** and ABA content **(B)** in six crops. Statistical analysis was carried out taking means of three replicates; different letters above bars showed significant differences as compared to control (Duncan's multiple range test *P* ≤ 0.05).

The application of calliterpenone modulated the plant's endogenous hormonal levels by increasing IAA and decreasing ABA contents (Figure 3). The maximum increases in IAA level were noticed with the concentration of 0.1 mM followed by 0.01 and 0.001 mM compared to control plants. However, a maximum reduction in ABA content was observed in 0.001 mM followed by 0.01 and 0.1 mM concentrations than control.

### Performance of calliterpenone in rice and wheat crops grown in farmers' fields

Results of 11 farmers' field trials from 3 districts (Lucknow, Sitapur, and Raebareli) showed that the application of calliterpenone resulted in an overall 11.93% increase in rice grain yield ranging from 3.48 to 19.63% (Table 4). This yield increase resulted from improvements in growth and yield contributing traits, *viz*., plant height (0.39%), tillering (3.34%), and panicle length (2.59%). In the case of wheat, an overall increase of 9.71% in grain yield, which ranged from 3.91 to 17.51% in different fields, was observed and an improvement in other growth and yield contributing traits, *viz*., plant height 2.79%, tillering 3.79%, and spike length 2.42%, were also recorded.

**Table 4:** Effect of calliterpenone on growth and yield of rice and wheat at farmers' field.

**Crops**	**Characters**	**% increase over control**	**Range**	***df***	***t* value**	***p* value**
Rice	Plant height	0.39	−0.40 to 1.19	20	0.47	0.65
	Tillers plant^−1^	3.35	1.39 to 5.71	20	2.26	0.04
	Panicle length	2.59	0.43 to 5.33	20	4.42	0.00
	Grain yield	11.95	3.48 to 19.63	20	4.32	0.00
Wheat	Plant height	2.791	0.75 to 4.38	20	2.22	0.04
	Tillers plant^−1^	3.791	2.22 to 5.75	20	1.53	0.14
	Spike length	2.423	0.49 to 3.50	20	1.80	0.09
	Grain yield	9.708	3.91 to 17.51	20	2.02	0.05

### Most effective doses

The effective doses of calliterpenone are presented in Table 5, which revealed that 0.001 mM of calliterpenone was the best effective dose for most crops except wheat, where a concentration of 0.01 mM was found to be the most optimal. The most appropriate stage for its application was found to be seed treatment, followed by a foliar spray at 30 DAP.

**Table 5:** Most effective concentration of calliterpenone in various crops.

**Crops**	**Effective concentration (mM)**	**Increased yield (%)**
Rice	0.001	28.89
Wheat	0.01	25.71
Tomato	0.001	28.63
Potato	0.001	32.46
Onion	0.001	20.63
Chickpea	0.001	26.08

### Safety studies

To test the formulation for its safety, the residue analysis of calliterpenone was carried out in the samples of rice for leaves, straw, grains, husk, and soil for all the treatments, and no traces of calliterpenone were recorded in any sample.

## Discussion

Plant growth regulators play a vital role in sustainable crop production by increasing plant morphological characteristics, including plant height, biomass, and fruiting. Organically produced foods are remarkably healthier, more attractive, and usually preferred by consumers (Hajšlová et al., 2005), but organic cultivation restricts the use of chemically synthesized plant growth hormones. Calliterpenone, a natural plant growth enhancer, therefore, might be a useful product for organic farming.

The plant growth-promoting activities of calliterpenone were found better than many cytokinins and gibberellic acid when tested during *in vitro* experiments (Singh et al., 2004; Goel et al., 2007). It has been reported earlier that calliterpenone possesses growth-promoting activities of microbes (Kalra et al., 2010; Patel et al., 2014), besides improving the yield and productivity of many plants (Haider et al., 2009; Singh et al., 2010, 2011; Bose et al., 2013; Maji et al., 2013; Zafar and Sangwan, 2019).

Seed yield is a very complex characteristic, which is the end product of different growth and developmental processes, influenced by several yield components (Chen et al., 2019). Generally, a higher number of effective tillers, large spikes, and more fertile seeds are closely associated with higher seed yield (Tiwari et al., 2011). It was clear that treating plants with calliterpenone results in increased plant height, tillering, spike length, and seeds spike^−1^ over the control, which might be associated with the growth-promoting properties of calliterpenone in stimulating cell division, elongation, and enlargement, which could have increased the total biomass of the plants (Bora and Sarma, 2006; Khassawneh et al., 2006).

Plant growth regulators have the potential to improve the actual crop yields once the crop growth is stimulated and the photosynthates are accumulated in the harvested products (Setia and Setia, 1990). A plant's improved vegetative growth and vigor are mostly responsible for enhanced seed yield because several photosynthesizing sites are affected by the initial growth stages. On the contrary, we found that although plant biomass increased at higher concentrations, seed yield was reduced. Earlier workers have also reported similar effects of calliterpenone on various crops (Goel et al., 2007; Haider et al., 2009; Singh et al., 2011). It was also found that foliar spraying of calliterpenone at 30 DAP was more effective than at 60 DAP. This effect may be due to the availability of plant growth substances at an appropriate time when the meristematic tissues of plants are maximally active. During active growth, dividing cells need more mobilization of nutrients triggered by the timely availability of phytohormones (Shah et al., 2006). However, active plant growth starts declining by 60 DAP; therefore, the spray of plant growth substance might have a limited role to play. Similarly, higher and/or repeated doses may cause desensitization of receptors, leading to no effect on plant growth. This higher dose-dependent receptor desensitization may be responsible for the terminating cell-signaling response at relatively higher doses of calliterpenone. However, after applications at doses below a threshold level, such receptors may again become sensitive (Xiao et al., 1999).

Foliar application of phytohormones affects the growth and yield of crop plants. It has been demonstrated that foliar treatments of IAA with appropriate amounts promoted plant growth and enhanced the yield of several crops like *Brassica* (Mir et al., 2020), *Guizotiaabys sinica* (Talukdar et al., 2022), *Linum usitatissimum* (Rastogi et al., 2013), and *Epipremnum aureum* (Di Benedetto et al., 2015), whereas its suboptimal application leads to a reduction in crop yield (Rastogi et al., 2013; Di Benedetto et al., 2015). In our studies, maximal benefits of the application of calliterpenone could be achieved with seed treatments. Cellular IAA-ABA interaction extensively regulates plant growth and development (Emenecker and Strader, 2020). Therefore, here we determined the content of IAA and ABA in calliterpenone-treated plants. Exogenous application of calliterpenone on the selected crops could influence the endogenous IAA and ABA levels (increased IAA and decreased ABA contents compared to control plants). Guzmán-Téllez et al. (2014) suggested that increased levels of endogenous IAA might result in an inhibitory effect; therefore, the optimal endogenous levels must be maintained for obtaining enhancement effects on various growth parameters. Similarly, owing to the endogenous accumulation of IAA, a significant increase in plant growth and biomass was observed, though the effect of these changes on seed yield is uncertain. Consequently, we found that the optimal level of IAA and ABA for maximum yield was a 0.001 mM concentration in most of the crops except wheat, where a concentration of 0.01 mM was found to be the most optimal. Recent studies have also shown that the exogenous application of hormones considerably increases endogenous IAA content and decreases ABA content in wheat plants (Yang et al., 2011; Cai et al., 2014).

Plant growth regulators have enormous potential for increasing crop yields, while their deployment and evaluation must be carefully designed in terms of optimal concentration, application levels, and species specificity (Khan and Mazid, 2018). The results of this study showed that the efficacy of calliterpenone, though not crop-specific, varies with the concentration, time of application, and methods of application. In rice, a maximum yield increase (28.89%) was observed through seed treatment with 0.01 mM calliterpenone followed by seed treatment with 0.001 mM (23.26%), both statistically at par and significantly higher than control. It was also observed that seed treatment followed by root treatment and a foliar spray at 30 DAP produced a lower yield than seed treatment with the same dose because seed treatment following root treatment and a foliar spray negatively impacted important yield components, *viz*., tillers plant^−1^ and seeds panicle^−1^. Though the concentration of 0.01 mM gave higher yields, we recommend a 0.001 mM dose of calliterpenone for rice seed treatment as it is more economical compared to 0.01 mM while producing at par yields. Similarly, in wheat, 0.1 mM concentration of calliterpenone applied through seed treatment and a foliar application at 30 DAP resulted in the highest yield (27.23%), followed by seed treatment and foliar application at 30 DAP with a 0.01 mM concentration (25.71%), which was at par with each other. This enhancement in yield was mainly associated with the greater number of seeds spike^−1^ compared to other treatments. Hence, we recommend a 0.01 mM concentration of calliterpenone in wheat applied through seed treatment followed by a foliar spray at 30 DAP. Tomato, potato, and onion are the three most consumed and produced vegetable crops in India. Production of these vegetables over the past few years has dramatically increased, making India the second largest vegetable producer in the world (Gulati et al., 2022). This achievement was made possible through improved varieties, modern farming practices, and the use of efficient fertilizers; besides this, PGRs are also becoming important in vegetable farming. In vegetable crops such as tomato, potato, and onion, the yield was significantly improved by applying calliterpenone with a 0.001 mM concentration. Seed treatment was found to be more effective in tomatoes and potatoes, whereas root treatment produced the maximum yield in onions. Moreover, the application of calliterpenone at earlier stages produced higher yields compared to later stages of crop growth, which is in conformity with the previous findings (Naeem et al., 2001; Shah et al., 2006). In chickpeas, foliar spray with 0.001 mM was found to be more effective than the treatment of the same dose through other application methods, so we recommend a 0.001 mM dose of calliterpenone as a foliar spray in chickpeas. These findings are in agreement with several recent studies that also found that foliar sprays of PGRs at limited concentrations significantly improve the growth as well as yield attributes in rice (Prajapati et al., 2021), wheat (Zhang et al., 2017; Dawar et al., 2021), potato (Thornton et al., 2013), tomato (Choudhury et al., 2013), onion (Ghani et al., 2021), and chickpea (Rafique et al., 2021).

## Conclusion

The plant growth-enhancing effects of calliterpenone (a phyllocladane diterpenoid) isolated from a medicinal plant *Callicarpa macrophylla* have been earlier demonstrated in many medicinal and aromatic plants, *viz., Pyrethrum, Mentha arvensis, Rauwolfia serpentina*, and *Artemisia annua* and found better than many well-established growth promoters such as gibberellins, auxins, and cytokinins. This encouraged us to study its effects on agriculturally important field crops (cereals: rice and wheat; vegetables: tomato, potato, and onion; and pulses: chickpea). Our results clearly illustrated that calliterpenone significantly enhances crop yields at a lower concentration of 0.001 mM in most crops except wheat, for which 0.01 mM was the most effective. Moreover, the exogenous application of calliterpenone could increase the endogenous level of IAA in all tested crops and reduce the content of ABA in many crops. Therefore, calliterpenone can be effectively used in augmenting the crop yields of agricultural/horticultural crops. Cost-effective and ready-to-use formulations of calliterpenone with a higher degree of purity enhance its potential for commercialization as a natural growth-promoting compound valuable for increasing crop yield and productivity. It will not only improve crop productivity and help in the upliftment of the socio-economic status of farmers, but it could also be helpful in solving world food problems. We firmly believe that it will sustainably increase crop yields and could be a vital input component of organic farming.

## Data availability statement

The original contributions presented in the study are included in the article/Supplementary material, further inquiries can be directed to the corresponding author/s.

## Author contributions

AK conceived the experiment and planned the methodology along with HS. AS and ST isolated the compound calliterpenone. PP did the experiment, collected data, and wrote the manuscript. AA helped in statistical analyses. SP and AT helped in manuscript preparation. AK corrected and finalized the manuscript. All authors reviewed and agreed on the final version. All authors contributed to the article and approved the submitted version.

## Funding

The Council of Scientific and Industrial Research, India, supported the study through Fast-Track Translational project FTT-5 (Calliterpenone for enhancing crop yields), and MLP-49 (Biostimulants for stress amelioration, enhanced plant productivity and soil health).

## Conflict of interest

The authors declare that the study was conducted in the absence of any commercial or financial relationships that could be construed as a potential conflict of interest.

## Publisher's note

All claims expressed in this article are solely those of the authors and do not necessarily represent those of their affiliated organizations, or those of the publisher, the editors and the reviewers. Any product that may be evaluated in this article, or claim that may be made by its manufacturer, is not guaranteed or endorsed by the publisher.
