# Corrigendum: Calliterpenone, a natural plant growth promoter from a medicinal plant *Callicarpa macrophylla*, sustainably enhances the yield and productivity of crops

**DOI:** 10.3389/fpls.2022.1055825

**Published:** 2022-11-25

**Authors:** Praveen Pandey, Shiv Shanker Pandey, Ashutosh Awasthi, Arpita Tripathi, Hemendra Pratap Singh, Anil Kumar Singh, Sudeep Tandon, Alok Kalra

**Affiliations:** ^1^ Microbial Technology Department, CSIR-Central Institute of Medicinal and Aromatic Plants, Lucknow, India; ^2^ Biotechnology Division, CSIR-Institute of Himalayan Bioresource Technology, Palampur, India; ^3^ Faculty of Education, Teerthanker Mahaveer University, Moradabad, India; ^4^ Biostatistics Department, CSIR-Central Institute of Medicinal and Aromatic Plants, Lucknow, India; ^5^ Herbal and Medicinal Products Division, CSIR-Central Institute of Medicinal and Aromatic Plants, Lucknow, India; ^6^ Process Chemistry and Chemical Engineering, CSIR-Central Institute of Medicinal and Aromatic Plants, Lucknow, India

**Keywords:** calliterpenone, plant growth regulators, IAA, ABA, yield contributing traits, sustainable crop production, medicinal plants

In the published article, there was an error in the legend for **Figure 1**: “Structure of calliterpenone and gibberellic acid”. The corrected legend appears below. There was also an error in **Figure 1**, Abbeokutone was mislabelled as “Gibberellic acid”.

The corrected **Figure 1** appears below.

**Figure 1:**
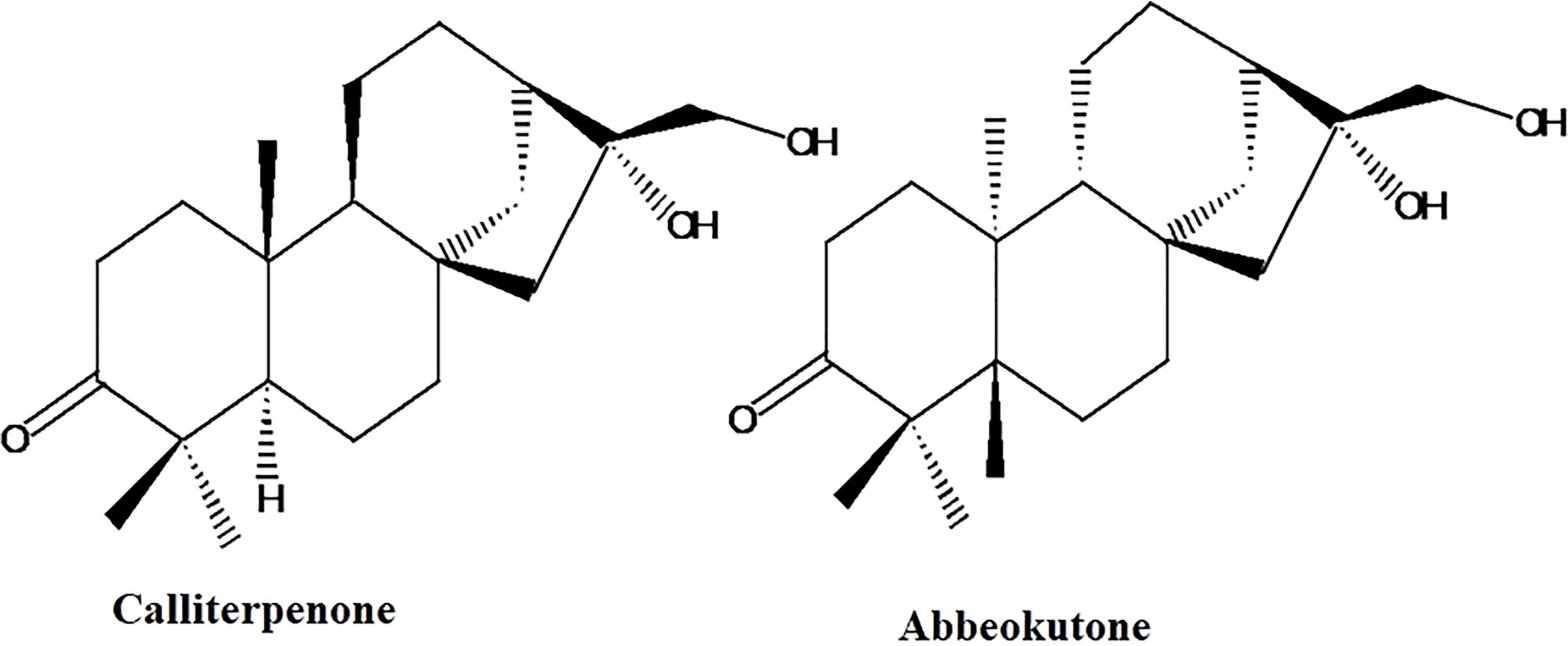
Structure of calliterpenone and abbeokutone.

In the published article, a compound name was misspelled in the Introduction. [“abeoketone”]. This sentence previously stated: “Calliterpenone (16α, 17-dihidroxy phyllocladane-3-one) (**Figure 1**), has a similar substitution pattern similar to the ent-kaurenoid compound “abeoketone” (16α, 17-dihidroxy kaurane-3-one), the precursor of gibberellins in the biosynthetic pathway (Liu et al., 2003; Bottini et al., 2004).”

The correct sentence appears as follows:

“Calliterpenone (16α, 17-dihidroxy phyllocladane-3-one) (**Figure 1**), has a similar substitution pattern similar to the ent-kaurenoid compound “abbeokutone” (16α, 17-dihidroxy kaurane-3-one), the precursor of gibberellins in the biosynthetic pathway (Liu et al., 2003; Bottini et al., 2004).”

The authors apologize for these errors and state that this does not change the scientific conclusions of the article in any way. The original article has been updated.

## Publisher’s note

All claims expressed in this article are solely those of the authors and do not necessarily represent those of their affiliated organizations, or those of the publisher, the editors and the reviewers. Any product that may be evaluated in this article, or claim that may be made by its manufacturer, is not guaranteed or endorsed by the publisher.

