# Supplementary material for: Calliterpenone, a natural plant growth promoter from a medicinal plant Callicarpa macrophylla, sustainably enhances the yield and productivity of crops
Source: Front Plant Sci. 2022 Sep 26;13:960717. doi: 10.3389/fpls.2022.960717 (PMC9549104; doi:10.3389/fpls.2022.960717)
Supplement: Supplementary file 1 [file Table_1.DOCX]

**Supplementary table S1 Percent yield increase over control through calliterpenone treatment in six crops**

| Crops | Treatments | Yield plot^-1^ (kg) | | Yield increase over control (%) | | Crops | | Treatments | | Yield plot^-1^ (kg) | | Yield increase over control (%) | |  |
| --- | --- | --- | --- | --- | --- | --- | --- | --- | --- | --- | --- | --- | --- | --- |
| 1. Rice | Control | | 3.46 + 0.06 | | - | | 2. Wheat | | Control | | 2.01 + 0.06 | | - | |
|  | ST1 | | 3.57 + 0.07 | | 3.07 | |  | | ST1 | | 2.44 + 0.05 | | 21.16 | |
|  | ST2 | | 4.46 + 0.13 | | 28.89 | |  | | ST2 | | 2.20 + 0.04 | | 9.59 | |
|  | ST3 | | 4.27 + 0.19 | | 23.26 | |  | | ST3 | | 2.26 + 0.03 | | 12.50 | |
|  | SRT1 | | 2.92 + 0.03 | | -15.71 | |  | | STA1 | | 2.56 + 0.04 | | 27.23 | |
|  | SRT2 | | 3.50 + 0.07 | | 1.16 | |  | | STA2 | | 2.53 + 0.02 | | 25.71 | |
|  | SRT3 | | 3.59 + 0.04 | | 3.64 | |  | | STA3 | | 2.30 + 0.01 | | 14.58 | |
|  | SRTA1 | | 3.09 + 0.09 | | -10.78 | |  | | STB1 | | 2.44 + 0.02 | | 21.29 | |
|  | SRTA2 | | 2.59 + 0.07 | | -25.28 | |  | | STB2 | | 2.34 + 0.06 | | 16.59 | |
|  | SRTA3 | | 3.83 + 0.04 | | 10.68 | |  | | STB3 | | 2.40 + 0.03 | | 19.57 | |
|  |  | |  | |  | |  | |  | |  | |  | |
| 3. Tomato | Control | | 8.05 + 0.12 | | - | | 4. Potato | | Control | | 6.37 + 0.12 | | - | |
|  | ST1 | | 9.70 + 0.28 | | 20.55 | |  | | ST1 | | 7.90 + 0.23 | | 24.08 | |
|  | ST2 | | 10.19 + 0.13 | | 26.59 | |  | | ST2 | | 8.73 + 0.08 | | 37.17 | |
|  | ST3 | | 10.35 + 0.14 | | 28.63 | |  | | ST3 | | 8.43 + 0.25 | | 32.46 | |
|  | SRTA1 | | 9.57 + 0.12 | | 18.94 | |  | | STA1 | | 7.33 + 0.22 | | 15.18 | |
|  | SRTA2 | | 9.28 + 0.38 | | 15.34 | |  | | STA2 | | 7.67 + 0.08 | | 20.42 | |
|  | SRTA3 | | 9.75 + 0.03 | | 21.10 | |  | | STA3 | | 6.80 + 0.07 | | 6.81 | |
|  | SRTB1 | | 9.96 + 0.14 | | 23.77 | |  | | STB1 | | 7.67 + 0.07 | | 13.86 | |
|  | SRTB2 | | 8.07 + 0.11 | | 0.22 | |  | | STB2 | | 7.60 + 0.17 | | 12.87 | |
|  | SRTB3 | | 9.77 + 0.26 | | 21.39 | |  | | STB3 | | 6.73 + 0.10 | | 5.76 | |
|  |  | |  | |  | |  | |  | |  | |  | |
| 5. Onion | Control | | 7.43 + 0.12 | | - | | 6. Chickpea | | Control | | 0.952 + 0.02 | | - | |
|  | RT1 | | 7.63 + 0.03 | | 2.69 | |  | | SP1 | | 0.988 + 0.02 | | 3.78 | |
|  | RT2 | | 8.83 + 0.05 | | 18.83 | |  | | SP2 | | 1.051 + 0.03 | | 10.35 | |
|  | RT3 | | 8.97 + 0.16 | | 20.63 | |  | | SP3 | | 1.201 + 0.02 | | 26.08 | |
|  | RTA1 | | 8.17 + 0.05 | | 9.87 | |  | | ST1 | | 0.987 + 0.02 | | 3.68 | |
|  | RTA2 | | 7.17 + 0.26 | | -3.59 | |  | | ST2 | | 0.958 + 0.02 | | 0.63 | |
|  | RTA3 | | 7.57 + 0.27 | | 1.79 | |  | | ST3 | | 1.098 + 0.02 | | 15.35 | |
|  | RTB1 | | 8.67 + 0.26 | | 16.59 | |  | | STA1 | | 0.822 + 0.02 | | -13.67 | |
|  | RTB2 | | 7.53 + 0.25 | | 1.35 | |  | | STA2 | | 1.152 + 0.06 | | 21.02 | |
|  | RTB3 | | 7.63 + 0.20 | | 2.69 | |  | | STA3 | | 1.145 + 0.03 | | 20.21 | |
